# Operative Treatment of Posterior Malleolar Fractures

**DOI:** 10.2174/1874325001711010732

**Published:** 2017-07-31

**Authors:** Xiaojun Duan, Anish R. Kadakia

**Affiliations:** 1 Center for Joint Surgery, Southwest Hospital, Third Military Medical University, Chongqing, 400038, P.R, China; 2 Department of Orthopedic Surgery, Northwestern University – Feinberg School of Medicine, Chicago, IL 60611, USA

**Keywords:** Ankle, Fracture, Operation, Approach, Treatment, Posterior Malleolar

## Abstract

Fractures of the posterior malleolus can occur in conjunction with fibular and medial malleolar fractures or in isolation. The indications for fixation of the posterior malleolus remain controversial except for the fragment sizes. A number of different surgical approaches and techniques for internal fixation of posterior malleolar fractures have been reported. Newer techniques such as direct exposure and plating of the posterior malleolus are chosen more frequently than traditional techniques of indirect reduction and percutaneous screw fixation. These attributes help to minimize the occurrence of postoperative complications.

## 1 INTRODUCTION

The annual incidence of ankle fractures is approximately 122-184/100,000 person years (1:800) [1]. Fractures of the posterior malleolus can occur in conjunction with fibular and medial malleolar fractures or in isolation [2-5]. Optimal treatment of ankle fractures with an associated posterior malleolus fragment is controversial [3, 6]. The purpose of this review is to discuss the current status of operative treatment of posterior malleolar fractures. Switaj *et ak.* has noted that 50% of operatively treated ankle fractures have a posterior malleolar component with 20% of cases having a posteromedial fragment, the so called “posterior pilon” variant [7].

## 2 CLASSIFICATION

Although many classification systems are proposed, three most widely used ones are the AO, Weber and the Lauge-Hansen classification [6, 8-10]. The Weber classification is based on the relationship of the level of the distal fibular fracture with the syndesmosis, in an attempt to quantify stability. The Lauge-Hansen classification is based on a cadaveric study involving two aspects the position of the foot at the time of injury and the direction of the applied deforming force. The study that Alexandropoulos *et al*. had done show that these three classification systems have in common a considerable interobserver variability deficiency which restricts their validity in selection of treatment options, prognosis and comparison between different materials [9]. Wang *et al*. recently reported a special type of trimalleolar fracture with the involvement of the entire posterior tibial plafond [11].

It is very difficult to accurately assess the shape and size of the posterior malleolar fragment, particular any involvement of the fibular notch, or the medial malleolus, on the basis of plain radiographs. Bartoníček *et al*. created a system for the classification of fractures of the posterior malleolus based on CT examination and takes into account the size, shape and location of the fragment, stability of the tibio-talar joint and the integrity of the fibular notch. It may be a useful indication for surgery and defining the most useful approach to these injuries [10]. Quantification of Three-Dimensional Computed Tomography (Q3DCT)-modelling is reliable to assess fracture characteristics of posterior malleolar fracture fragments. Mangnus *et al*. felt that morphology might be more important than posterior malleolar fracture size alone for clinical decision making [12]. Currently, there is no accepted method by which to classifyposterior malleolar fracture that aids in identifying those fracture patterns that require operative intervention.

## 3 INDICATIONS

The indications for fixation of the posterior malleolus remain controversial. The current indications are varied and evolving and include fractures involving >25% to 33% of the articular surface, displacement>2 mm, ankle instability with concomitant syndesmotic injury, and persistent posterior subluxation of the talus [2, 13-16].

This value has been questioned more recently as studies have demonstrated the importance of even small posterior malleolar fragments to ankle stability, and surgical indications have expanded [17]. The historic criteria based on size truly underestimate the number of fractures that require operative intervention. Failure to fix a mal-reduced posterior malleolus may result in late posterolateral subluxation of the fibula and failure of syndesmotic fixation. Failure to restore the articular congruity of the articular surface, including the posterior malleolus within 2mm is associated with worse functional outcomes at 1 years compared to those patients who were congruent [18]. Additionally, a significant incidence of radiographic arthritis was demonstrated if a stepoff of greater then 1mm was noted with regards to the posterior malleolus regardless of the fragment size [13].

In a prospective study of outcomes of syndesmotic injuries with posterior malleolar fractures, Miller *et al*. found that posterior malleolar fixation was equivalent to fixation with syndesmotic screws or combined fixation. The results of these studies would lead to the conclusion that even small posterior malleolar fractures should be repaired in ankle fractures with syndesmotic disruption [19]. Anatomic reduction of the posterior malleolus additionally recreates the incisura fibularis and should minimize the risk of posterior translatory malreduction of the syndemosis as the PITFL is intact in all cases of a posterior malleolar fracture. Some authors have gone so far as to say ligamentous repair of the PITFL is critical when operatively treating ankle fractures [20]. Although, it is a very novel concept and is not advocated by the authors as routine practice, the concept of anatomic restoration of the ligamentous stability of the ankle is increasing.

## 4 TIMING

Many surgeons will perform surgical treatment within two weeks, usually the skin of ankle appears wrinkled which mean the soft tissue swelling subsided [3, 21]. Information about the influence of delayed surgery on infectious wound complications is ambiguous. The clinical audit was performed by Schepers *et al*. The systematic review of the literature showed a difference in wound complications of 3.6% (early surgery) versus 12.9% (late surgery) (p < 0.0001). A delay in surgery is associated with a significant rise in infectious wound complications, which significantly lowers outcome and patient satisfaction. These fractures should preferably be treated within the first day [22].

## 5 TECHNIQUE

A number of different surgical approaches and techniques for internal fixation of posterior malleolar fractures have been reported [21, 23-31]. Mast *et al*. reported that indirect reduction and stabilization with anteroposterior (AP) screws and that remains the early common method [14]. Occasionally, the posterior fragment reduces simultaneously when the lateral malleolus is reduced because of their respective attachments to the posteroinferior tibiofibular ligament (PITFL). This fragment can then be fixed with lag screws inserted from anterior to posterior. However, the surgeon must be sure that the fragment is both reduced and that the screw is actually capturing the posterolateral fragment. Patel *et al* present a case of symptomatic tibial nerve impingement (entrapment) caused by an AP screw used to fix the posterior malleolus in a trimalleolar ankle fracture [32]. Avoidance of this complication, they advise to make fixation using a posterolateral approach the optimal method for treating these fractures. The authors’ preference is to directly visualize the reduction and place fixation from posterior to anterior to ensure that appropriate compression of the fracture is achieved to restore articular congruity.

This expected reduction is not likely if the ankle is not being fixed acutely because of the interposition of organized hematoma or callus. If direct exposure of the fragment is necessary, the posteromedial approach has been recommended twenty years ago [33, 34]. This allows fixation of the medial and posterior malleolus through the same incision. With posteromedial approach, it may access the posterior malleolus by incising the sheaths of the tibialis posterior and flexor digitorum longus tendons and retracting them anteriorly [35]. A medial approach for a typically posterolateral fragment still would seem suboptimal. An extensile posteromedial approach with dislocation of the talus laterally and complete release of soft-tissue attachments to the posterior malleolus has also been described. This seems overly aggressive and can compromise syndesmotic integrity. The limited visualization of the posterior malleolar fragment afforded by this exposure has led other authors to describe different techniques to facilitate anatomical reduction. Other options include arthroscopically assisted reduction [36] and the lateral transmalleolar approach [37]. It is very difficult to get an anatomical reduction of the posterior malleolus fragment using a lateral transmalleolar approach, however, because the PITFL is attached to the unreduced fibula. But Kim *et al*. reported that Lateral transmalleolar approach and miniscrews fixation for displaced posterolateral fragments of posterior malleolus fractures in adults and the result was satisfied [31].

**Fig. (1):**
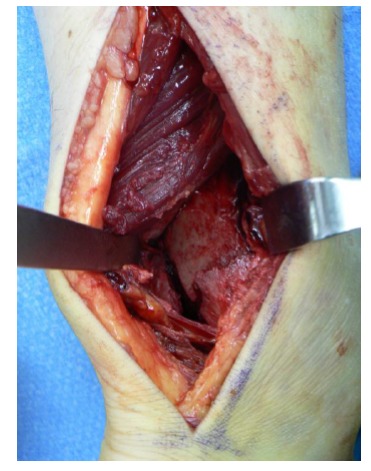
View of the posterior fragment in the interval between the peroneal tendons and the flexor hallucis longus.

**Fig. (2):**
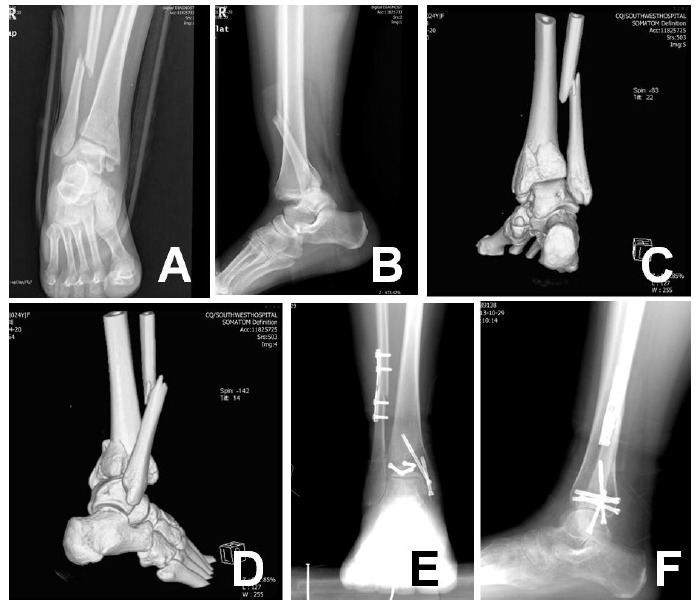
Case 1: (**A** and **B**) Anteroposterior and lateral radiographs showing a displaced trimalleolar fracture, (**C** and **D**) Three-Dimensional Computed Tomography, (**E** and **F**) Postoperative anteroposterior and lateral radiographs.

**Fig. (3):**
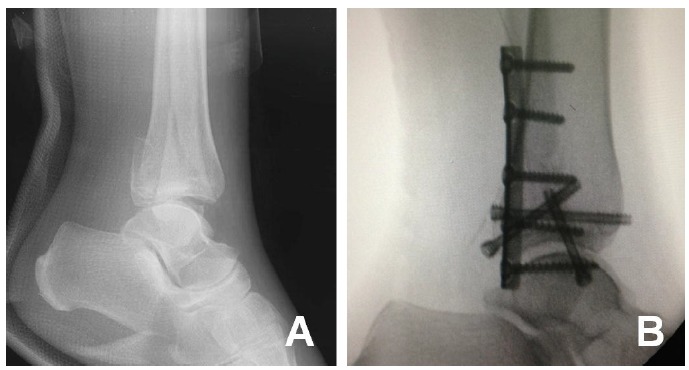
Case 2: (**A**) Lateral radiographs showing a displaced trimalleolar fracture, (**B**) Intra-operative lateral radiographs.

The posterolateral approach has been described in the literature and has received much attention [38-42]. Surgery is performed in the prone position with a bump under the ipsilateral hip and the posterolateral approach is performed. Most surgeons like to choose the incision just between the peroneal tendons and Achilles tendon (Fig. **1**), but Talbot *et al*. did the longitudinal incision is placed just medial to the posterior border of the fibula [38]. The lesser saphenous vein and sural nerve are identified and protected. The sural nerve courses from medial to lateral and crosses the lateral border of the Achilles tendon on average 9.8 cm proximal to its insertion in the calcaneus. At a point 7 cm proximal to the tip of the lateral malleolus, the nerve is on average 26 mm posterior to the edge of the fibula [43]. Wang *et al*. reported that the distance was 7.2 ± 4.1 mm between the sural nerve and the posterior section of the fibula [26]. The surgeon must be aware that the anatomy of the sural nerve is highly variable and the best way to protect it and avoid nerve injury and neuromas is to perform meticulous blunt dissection in the subcutaneous tissue. The lateral malleolus may be fixed by elevating the peroneal tendons laterally or medially through the same skin incision [41]. The fibular fracture is classically fixed with a lag screw and an antiglide plate (Fig. **2**), but the fixation construct may vary according to fracture pattern or comminution. Although the lateral aspect of the fibula can be visualized through this approach, placement of the screws requires significant soft tissue retraction and is difficult. Therefore, posterolateral plate placement is ideal. Plate fixation of the fibula should be avoided until the posterior malleolus is stabilized in order to ensure appropriate visualization of the distal tibial articular surface (Fig. **3**).

A second interval is then exploited within the wound. The flexor hallucis longus is lifted off the posterior tibia allowing access to the posterior malleolus. Care is taken to preserve the PITFL attachment to the fragment and the joint capsule, which means the fragment should be booked open from medial to lateral for joint inspection [28]. Blood is supplied to the posterior tibia by the perimalleolar arterial ring from which fine arterial branches penetrate the bone 2.5-5 cm proximal to the joint line. The fragment, depending on its size, is fixed with screws from the small or mini fragment set. A small buttress plate can also be used to supplement fixation. This is some surgeon’s preferred method of fixation. Small interposed fragment are reduced if possible, however, removal of loose bodies may be required if they cannot be reduced and fixed. After “booking” the fracture open, larger articular fragments can be stabilized with K-wires that are direct out the anterolateral ankle, ensuring the wire is flush to the posterior bone. This will allow reduction and fixation of the remaining posterior malleolus, “closing the book”. The K-wire can then be removed anteriorly following final fixation of the posterior malleolus.

Addressing the postero-medial malleolar fracture can be done through the same approach, although visualization is difficult. Making the approach immediately lateral to the Achilles tendon can facilitate visualization and reduction of the entire posterior malleolus and the lateral malleolus [44]. The posterior tibial tendon must be elevated off the tibia in order to allow for appropriate plate placement. Alternatively, a separate posteromedial incision can be made immediately anterior to the posterior tibial tendon. The sheath of the posterior tibial tendon is elevated and the tendon retracted laterally. This approach allows visualization of the fracture, facilitates plate placement, and avoids the neurovascular bundle. In order to decrease irritation of the posterior tibial tendon from hardware placement, either headless screws or avoidance of screw placement within the area of the plate that lies within the posterior tibial tendon groove should be undertaken.

Lastly, the medial malleolus can be addressed through a standard medial incision or distal extension of the posteromedial incision if a posteromedial fracture is present. Access to the medial side is slightly more challenging in the prone position, as compared with the supine position, because of the leg's propensity to rotate externally. Fixation of the medial side is carried out classically with two 3.5 mm lag screws, but again may vary according to fracture pattern [38]. In many cases of a posteromedial fracture component, only the anterior colliculus of the medial malleolus is fractured. In this case use of a 2.0 or 2.4mm screw may be required given smaller width of the fragment.

Franzone *et al*. reported other details about surgical technique [28]. Using the intact PITFL as a hinge, the posterior malleolar piece is then rotated laterally to provide access to the impacted bone anterior to the fracture line. This area of the plafond is then disimpacted, and allograft bone may be placed into any resultant defect. The posterior malleolar fragment is then anatomically reduced and provisionally fixed with a Kirschner wire. Once the fractured malleolus is replaced, the joint reduction is no longer visible, and so it is the posterior tibial cortex that provides direction as to the appropriate positioning of the posterior malleolus. The posterior malleolus was reduced directly and provisionally fixed with K wires. It was then fixed with either a small fragment T plate or 1/3 tubular plate applied in a buttress technique [17].

Heim found the posterolateral approach especially useful for patients with smaller, posterior fragments [45]. Franzone *et al*. reported the technique that the posterior malleolus is fixed prior to the distal fibula. Since internal fixation devices on the fibula would potentially block radiographic visualization of the reduction of the posterior malleolus [28]. Choi *et al*. described the single oblique posterolateral approach that was more closed with fibula than others and found that it had the potential to decrease the incidence of sural nerve injury because of the smaller incision size [30]. The fracture was openly reduced and fixed through a combined operative approach (posterolateral and posteromedial) when Wang *et al*. performed surgery for the entire posterior malleolar fragment [11].

In our experience, we have found that the posterolateral approach has several advantages. The main advantage is that it allows a direct inspection and reduction of the posterior fragment. Anatomical reduction of articular surfaces is a basic principle in fracture surgery, and this approach certainly promotes that goal. In the case of delayed surgery, the fracture can be cleaned out directly, removing interposed callus, once again promoting an anatomical articular reduction. And the direct visualization allows for the joint to be inspected for osteochondral fragments, talar chondral damage or impaction injury. With this exposure, the surgeon can choose to supplement fixation of the posterior malleolus with a buttress plate, also a basic fixation principle in a weight-bearing joint that will experience axial load or shearing forces during weight bearing. In addition, this is the exposure of choice for the use of an antiglide plate for fibular fixation. Such a posterolateral fibular construct has been shown in biomechanical studies to be superior to the more commonly used lateral plate. Soft-tissue coverage for the plate is also enhanced in the posterior fibular position.

There are some drawbacks to the posterolateral approach. It had the potential to cause the incidence of sural nerve injury. It is true that the prone position can make ORIF of the medial malleolus more challenging. Furthermore, in cases with associated forefoot or talus fractures or anterior syndesmotic injuries, moving the patient to a supine position will be necessary as these injuries cannot be addressed through this incision or in the prone position.

## 6 FIXATION

Generally, posterior malleolar fragments are fixed either with percutaneous anterior to posterior (AP) screws or through a posterolateral approach using screws and/or a buttress plate [15, 17, 42, 46, 47]. Fixation with AP screws relies on reduction of the posterior malleolus through ligamentotaxis of the posterior inferior tibiofibular ligament with reduction of the fibula, whereas fixation through a posterolateral approach allows direct reduction of the fracture. In essence, the posterior malleolar fragment is an AO type B articular injury. As a principle, the majority of AO type B injuries in other areas are treated with buttress plating rather than screw fixation from the opposite side. OʼConnor *et al*. had done the retrospective comparative study [17]. They found that patients with trimalleolar ankle fractures in whom the posterior malleolus was treated with posterolateral buttress plating had superior clinical outcomes at follow-up compared with those treated with AP screws.

Gardner *et al*. surveyed 401 orthopaedic surgeons regarding preference and indications for choice of fixation between posterolateral plating and AP screws [15]. Seventy-two percent of trauma-trained orthopaedic surgeons preferred direct open reduction versus 53% of foot and ankle trained and only 39% of surgeons who were not subspecialty trained in trauma or foot and ankle. Despite the majority of trauma-trained surgeons choosing a direct open approach, only 56% chose posterolateral plating as their preferred method of fixation.

## 7 REHABILITATION

This is consistent with modern rehabilitation principles [3, 48, 49]. The postoperative protocol is to remain in the initial splint for 2 weeks and then transition to a boot for weeks 2-6 while allowing range of motion (ROM) and stretching exercise. Patients are instructed to begin weight bearing at 6 weeks and with full weight bearing by 12 weeks [17]. Wang *et al*. reported that range of motion exercises were started in reliable patients after the first 3 weeks [11]. Partial weightbearing was allowed after 6 weeks, then clinical assessment of fracture-healing was made when no pain or tenderness with weightbearing or walking. Full weightbearing was restricted for about 3 months postoperatively.

Firoozabadi *et al*. believe that a certain subset of surgical ankle fracture patients can be made weight-bearing as tolerated immediately following surgery [50]. Immediate weight-bearing as tolerated allows patients to return to ambulation and activities of daily living faster and may facilitate rehabilitation. Only 1/26 patients was noted to have loss of fixation. This was found at the 6-week follow-up and was attributed to a missed syndesmotic injury. Smeeing *et al*. had done the meta-analysis show that following ankle surgery [51], 1) active exercises accelerate return to work and daily activities compared to immobilization, 2) early weight-bearing tends to accelerate return to work and daily activities compared to late weight-bearing. They found that active exercises in combination with immediate weight-bearing may be a safe option.

A cross sectional expert opinion survey was administered to members of the AOFAS as well as OTA to determine how long they would instruct patients to be non-weight bearing after open reduction and internal fixation of ankle fractures. Seven hundred and two surgeons (31%) responded to the survey. There is significant variation among orthopaedic surgeons when selecting period of non-weight bearing after fixation of ankle fractures, with both injury pattern and medical comorbidity playing a role in decision of time to keep patient non-weight bearing. The average time of non-weight bearing selected varied from 4.9 (± 3.1) weeks for in young, healthy patients with SER4 equivalent injuries to 7.6 (± 6.0) weeks for older patients with medical comorbidities with trimalleolar fractures [52].

## 8 COMPLICATION

Little *et al*. had done the study to determine the complication rate for ankle fractures treated through the posterolateral approach [27]. They found there were 11 minor wound related complications (9.8%) and 3 major wound complications (2.7%), 1 of which required a split thickness skin graft. The overall postoperative wound infection rate was 4.4% (5 of 112); 2 patients required hardware removal due to deep infection. Of patients, 7% (8 of 112) reported symptomatic lateral sided hardware and thus underwent removal of implants. The overall reoperation rate was 12.5%. The complication rate was 23%. No patients experienced loss of reduction. They reported that there was only 1 case of superficial peroneal nerve (SPN) injury noted in their study. Their approach centered anterior to the sural nerve and parallel to its path decreasing the risk for injury [27]. Choi *et al*. reported the surgery was complicated by skin necrosis around the incision in 2 (4%) patients and sural nerve damage in 2 (4%) patients [30]. Huang *et al*. reported that the excellent-to-good rate was 93.8% and just one patient had a superficial infection [21]. The many results show that the posterolateral approach to the ankle is a valuable approach for the treatment of posterior malleolar fracture [21, 30, 32, 40, 42, 53, 54].

The most common cause of ankle joint arthritis is posttraumatic with estimated incidence to be in the region of 70%; rotational injuries being the commonest cause. The risk of venous thromboembolism (VTE) is reported to be low in patients with ankle fracture; deep vein thrombosis (DVT) (0.12%) and pulmonary embolism (PE) (0.17%) [55].

## 9 PROGNOSIS

Ankle fractures involving the posterior malleolus have been shown to have worse outcomes compared with ankle fractures without posterior malleolar involvement [56]. The most common age groups affected are young active patients, sustaining high energy trauma and elderly patients with comorbidities. Both these groups pose unique challenges for appropriate management of these injuries. Young patients are at risk of developing posttraumatic osteoarthritis, with a significant impact on quality of life due to pain and impaired function. Elderly patients, especially with poorly controlled diabetes and osteoporosis are at increased risk of wound complications, infection and failure of fixation. In the most severe cases, this can lead to amputation and mortality. Therefore, individualized approach to the management of AF is vital [55]. Elderly and diabetic patients are at particular risk of complications. Routine removal of metalwork is not advised in the asymptomatic patients.

Forberger *et al*. found that direct exposure and reduction reduced the rate of poor clinical outcome [39]. This might be an effect of the debridement and better reduction of the fragment. The mobility evident at the final follow-up underlined the good anatomic results with only minor limitations compared to the unaffected side. While there was significantly less flexion and pronation compared to the unaffected side, the extension and supination were not significantly restricted. In their opinion the size of the posterior malleolar fragment is not the most important factor affecting outcome. More important is whether the stability of the joint surface can be restored, especially in ankle fractures with total dislocation [39].

Abdelgawad *et al*. performed the posterolateral approach for treatment of posterior malleolus fracture of the ankle [42]. A total of 12 consecutive patients were follow-up. No deep infection or wound dehiscence occurred. Ten patients had adequate (< 2-mm displacement of the articular surface) radiologic reduction at the final follow-up visit. There were 2 cases of 2 mm or more of articular surface displacement at the final follow-up visit.

Clinical studies have been inconclusive regarding optimum treatment of posterior malleolus fractures [7, 14, 21, 39, 42, 57-61]. While some authors have found no differences regarding clinical outcomes and ankle stability in posterior malleolus fracture with or without posterior fixation, others have found that large fragments undergoing reduction and fixation yielded better results than those without fixation. Furthermore, non anatomical reduction of posterior malleolus fractures has been shown to lead to worse outcomes than nonoperative treatment. Other studies have shown ankle fractures with involvement of the posterior malleolus lead to poorer outcomes even when the fragment is small, with worse outcomes as fragment size increases. Some studies have suggested that outcomes correlate to the extent of the injury and are not necessarily affected by the posterior malleolus fragment alone, but rather by concomitant injury to articular cartilage, ligamentous structures, talar vascularity, and the presence of osteochondral fragments [15].

## CONCLUSION

In conclusion, the posterior malleolus fracture is a common injury with potentially significant morbidity associated with it. Most notably, factors include fragment size most impacted surgical indications. Newer techniques such as direct exposure and plating of the posterior malleolus are chosen more frequently than traditional techniques of indirect reduction and percutaneous screw fixation. These attributes help to minimize the occurrence of postoperative complications.
